# Thermal Interaction Mechanisms of Ammonium Perchlorate and Ammonia Borane

**DOI:** 10.3390/molecules30132680

**Published:** 2025-06-20

**Authors:** Yunlong Zhang, Rui Pu, Shaoli Chen, Qilong Yan

**Affiliations:** National Key Laboratory on Solid Rocket Propulsion, Northwestern Polytechnical University, Xi’an 710072, China

**Keywords:** ammonia borane, ammonium perchlorate, NH_3_BH_2_ClO_4_, integrated design of fuel oxidant

## Abstract

Ammonia borane (AB), with a theoretical hydrogen content of 19.6 wt%, is constrained by its low crystalline density (0.758 g/cm^3^) and poor thermal stability (decomposing at 100 °C). In this study, AB/ammonium perchlorate (AP) composites were synthesized via freeze-drying at a 1:1 molar ratio. The integration of AP introduced intermolecular interactions that suppressed AB decomposition, increasing the onset temperature by 80 °C. Subsequent vacuum calcination at 100 °C for 2 h formed oxygen/fuel-integrated ammonium perchlorate borane (APB), which achieved decomposition temperatures exceeding 350 °C. The proposed mechanism involved AB decomposing into borazine and BN polymers at 100 °C, which then NH_3_BH_2_^+^/ClO_4_^−^ combined to form APB. At 350 °C, APB underwent the following redox reactions: 4NH_3_BH_2_ClO_4_ → N_2_↑ + 4HCl↑ + 2B_2_O_3_ + N_2_O↑ + O_2_↑ + 7H_2_O↑ + H_2_↑, while residual AP decomposed. The composite exhibited improved density (1.66 g/cm^3^) and generated H_2_, N_2_, O_2,_ and HCl, demonstrating potential for hydrogen storage. Additionally, safety was enhanced by the suppression of AB’s exothermic decomposition (100–200 °C). APB, with its high energy density and thermal stability, was identified as a promising high-energy additive for high-burning-rate propellants.

## 1. Introduction

Solid propellants served as the power source for various solid motors used in missiles, weaponry, and spacecraft for space launches. The energy performance of propellants determines their effective range and deterrence capability. Consequently, recent research in the field of high-energy solid propellants has focused primarily on exploring and developing new high-energy-density materials and enhancing the energy content of existing energetic materials. Hydrogen storage fuels [1] release hydrogen upon thermal decomposition, which reduces the average molecular weight of combustion products, thereby increasing the specific impulse (*I*_sp_) of the propellant. Therefore, incorporating hydrogen storage materials into propellant compositions could effectively enhance the energy level of solid propellants [1]. Chemical hydride hydrogen storage materials mainly refer to hydrogen combined with other solid materials, such as metals, through ionic or coordination bonds. Chemical hydrogen storage is the most extensively researched and promising method of hydrogen storage. It is broadly categorized into metal-based and non-metal-based hydrogen storage materials. Metal-based hydrogen storage fuels primarily include metal hydrides such as aluminum hydride (AlH_3_) and metal borohydrides [2,3,4,5,6,7,8,9,10,11,12,13,14]. However, AlH_3_ is unstable and reacts with water and moist air to produce hydrogen, while borohydrides have high thermodynamic stability, which poses challenges in hydrogen release during use.

Ammonia borane (AB), as a non-metal hydrogen storage fuel [7,15], has garnered significant attention due to its high chemical hydrogen storage capacity (theoretical hydrogen content of 19.6 wt%). The first weight loss of AB occurs during the melting process, which is caused by the thermal dehydrogenation of AB, accompanied by the generation of foamy products [16]. This exothermic process is very rapid and reaches the maximum decomposition rate between 107 and 125 °C, depending on factors such as heating rate, impurities, and particle size [17,18,19,20,21,22,23]. The second decomposition step occurs at approximately 130 °C, accompanied by hydrogen release [24]. In the temperature range of 95 °C to 177 °C, due to the slow decomposition reaction, two exothermic peaks are typically observed on the DSC curve. After the two-step decomposition, a polymeric residue (BNH)_x_ is obtained, which further dehydrogenates at temperatures well above 500 °C [17,18,19,20,21,22,23]. When the temperature is raised to 1170–1500 °C, the product transforms into hexagonal boron nitride crystals.

Other by-products have been detected through TGA-MS or TGA-FTIR techniques, primarily borazine (B_3_N_3_H_6_) [25,26]. During the first decomposition step, approximately 7 wt% of B_3_N_3_H_6_ is formed within the temperature range of 100–200 °C, with the remainder being produced in the second step [27]. Other minor gaseous products included diborane, ammonia, H_2_N-BH_2_, H_2_BNH_2_BH_3_, and sublimated AB.

From these observations, three distinct reaction pathways for the thermal decomposition of AB have been summarized. The hydrogen release process of AB encompasses the three stages mentioned above, accompanied by the release of hydrogen gas and impurities, as well as the generation of (BNH)_x_, which is mainly produced during the second stage. Due to the polarity differences between the B-H and N-H bonds in AB, the thermal decomposition of AB proceeds through anisotropic interactions and intermolecular dehydrogenation bonding [28,29,30].

In the initial stages of decomposition, chain products such as BH_2_, BH, and BH_3_ are rapidly formed. These compounds subsequently transform into diammoniate of diborane (DADB) and H_2_ [30,31,32,33,34,35,36]. During this process, by-products such as ammonia diborane (ADB) are also produced, accompanied by the release of a significant amount of hydrogen. The chain substances undergo autocatalytic reactions to form polyaminoboranes (PABs). Under the catalysis of AB and NH_2_BH_2_, PABs participate in two competing reactions: propagation to form acyclic PABs or cyclization. Due to subsequent cyclization and cross-linking reactions, the formation of cyclic PABs requires higher temperatures, and these reactions also lead to the formation of borazine (BZ) and polyborazylene (PBZ). The presence of NH_3_ can facilitate the reverse synthesis of AB from NH_3_BH_2_(μ-H)BH_3_ [37,38]. DADB is the initial product of further decomposition of cyclic products of AB and can also be formed through dehydrogenation bonding between AB molecules. The ring-opening reaction of DADB leads to its gradual conversion into BZ, ultimately yielding polyborazylene (PBZ).

Notably, some progress has been made in the research on AB with oxidizers. Studies found that when AB is composited with AP, the presence of NH_4_^+^ ions allows the thermal reaction of AB to follow an alternative pathway, generating [NH_3_BH_3_NH_3_]^+^[ClO_4_]^−^ instead of DADB [28,29,30,31,32,33,34,35,36,39,40].

AB is an attractive hydrogen storage material, not only due to its high theoretical hydrogen storage capacity but also because of its easier hydrogen release, which outperforms other hydrogen storage materials such as AlH_3_ and metal borohydrides [8,9,10,11,12]. However, its low density and poor thermal stability limit its applications. When AB is composited with AP, the presence of NH_4_^+^ ions enables the thermal reaction of AB to follow an alternative pathway, generating [NH_3_BH_2_NH_3_]^+^[ClO_4_]^−^ instead of DADB [28,29,30,31,32,33,34,35,36,40], which indicates the potential formation of ammonium perchlorate borane (APB).

Guillaume [41] successfully synthesized high-energy-density ammonia dinitroamine borane through the reaction of dinitroamine with ammonia borane. This synthesis method provides a research idea to solve the problem of low density and low thermal stability of AB.

In view of the poor thermal stability and low density of AB, the researchers used AB, AP, graphene (GA), and graphene oxide (GO) as raw materials, weighed AB and AP according to the molar ratio of 1:1, and prepared AB/AP composite particles by the direct freeze-drying method. At the same time, 2 wt% GA or GO was added to prepare carbon material modified AB/AP-GA and AB/AP-GO. Then, AB/AP(Δ), AB/AP-GA(Δ), and AB/AP-GO(Δ) were synthesized by thermal reaction of the three under vacuum. The specific formula is shown in Table 1 and Table 2. APB was detected in three kinds of calcined AB/AP. The pyrolysis mechanism and physicochemical properties of APB were systematically studied [28,29,30,31,32,33,34,35,36,40]. The prepared oxidant/fuel integrated APB has high thermal stability and high density. APB has excellent energy performance and thermal stability, and is considered to be a promising high-energy additive for high-burning-rate solid propellant.

In response to AB’s poor thermal stability and low density, researchers prepared AB/AP composite particles via direct freeze-drying and thermally reacted them under vacuum to synthesize APB. The pyrolysis mechanism and physicochemical properties of APB were systematically studied. The resulting oxidant/fuel-integrated APB exhibited both high thermal stability and increased density. Due to its superior energy performance and excellent thermal stability, APB was considered a promising high-energy additive for high-burning-rate solid propellants.

## 2. Results

### 2.1. Microscopic Morphology and Elemental Compositions

**Figure 1:**
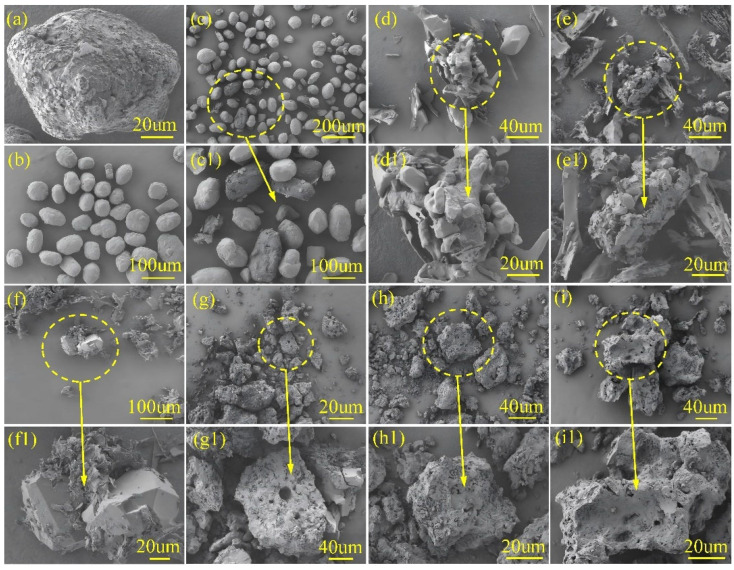
SEM images of AB/AP composites: (**a**) AB, (**b**) AP, (**c**) mechanical mixture AB/AP, (**d**) AB/AP, (**e**) AB/AP-GA, (**f**) AB/AP-GO, (**g**) AB/AP(Δ), (**h**) AB/AP-GA(Δ), and (**i**) AB/AP-GO(Δ).

SEM morphological analysis revealed that AB particles exhibited irregular shapes, with most particles measuring approximately 100 μm and displaying numerous surface pits. In contrast, AP crystals predominantly adopted regular cubic or spherical morphologies, with an average particle size similarly around 100 μm. Figure 1 shows the morphology of the AB/AP mechanical mixture, where well-defined AP particles and AB particles with surface cavities and cracks were observed, both with particle sizes close to 100 μm. The AB/AP crystal morphology, as depicted in Figure 1, presented a fragmented structure alongside agglomerates of regular crystals. Notably, small, well-defined particles appeared enveloped by fibrous, cotton-like particles, with an average crystal size of only 30 μm. AB/AP-GA displayed agglomerative growth characteristics, featuring elongated graphene strands embedded within the composite particles. The overall particle morphology resembled that of AB/AP, with agglomerated particles measuring between 30 and 40 μm. Similarly, AB/AP-GO demonstrated crystal agglomeration but with larger dimensions, accompanied by numerous small, flake-like crystals filling the interparticle gaps. Comparative analysis indicated that the addition of GA or GO eliminated the cotton-like coating observed in AB/AP, instead promoting the growth of fine, fragmented structures interspersed among larger crystals. This observation suggested that the presence of GA and GO modified the AB/AP crystallization process, resulting in altered agglomeration morphologies. AB/AP(Δ) formed irregular bulk particles approximately 100 μm in size, containing abundant internal pores. AB/AP-GA(Δ) exhibited a morphology similar to APB, with comparable particle sizes around 100 μm. AB/AP-GO(Δ) similarly consisted of large, irregular particles with surface pores measuring about 100 μm. These surface pores originated from hydrogen evolution during the thermal treatment process, after which perchlorate ions combined with the foamed boron-nitrogen polymer to form the ammonia perchlorate borane derivative, APB.

Table 3 presents the elemental composition of the AB/AP composite. The elemental analysis results indicate that thermal treatment led to a reduction in the nitrogen (N) and hydrogen (H) content within the composite, which is attributed to both the release of hydrogen gas during the thermal treatment process and the generation of NH_3_, N_2_, and trace amounts of nitrogen oxides through AP’s redox reaction. The escape of these gaseous products consequently caused a significant decrease in the N and H content in the calcined product.

The oxygen (O) content of AB/AP-GO and AB/AP-GO(Δ) decreased significantly compared with the theoretical results. This is due to a large number of bubbles emerging from the sample with added GO during the preparation process. It is speculated that the poor compatibility between GO and AB in water may lead to the hydrolysis reaction of AB showing strong reducibility, and the oxidation–reduction reaction with a small amount of ClO_4_^−^ may lead to the reduction of the measured O content of AB/AP-GO and AB/AP-GO(Δ).

### 2.2. Chemical Bond Structure and Energetic Properties

**Figure 2:**
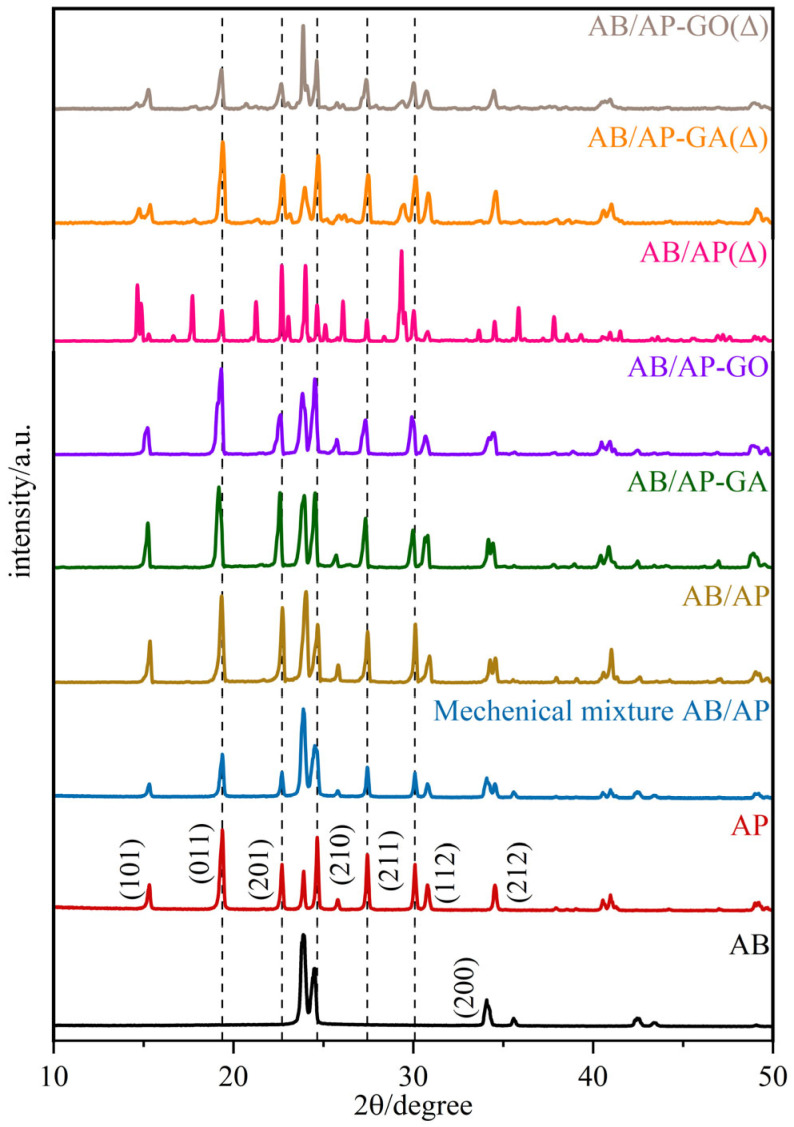
XRD pattern of composite particles.

Simultaneously, the XRD diffraction patterns (Figure 2) revealed that the diffraction peak positions of both AB/AP composite particles and modified AB/AP composites were essentially identical to those of the prepared mechanical mixture, differing only in peak intensity. Table 4 presents the unit cell parameters of the crystalline samples. The data indicate that the crystal structures of the three AB/AP composites underwent significant changes: while both AB and AP exhibit an orthorhombic system, the composite particles adopted a monoclinic system. Although the three composites share the same crystal system, AB/AP-GO belongs to a different space group compared to the other two. These results confirmed that the characteristic diffraction peaks of AB and AP remained present in the composites modified with GA or GO, but the incorporation of graphene-based materials did not facilitate the formation of new crystal planes between AB and AP. Instead, all composite particles retained a mechanically mixed co-particle morphology. The XRD powder diffraction patterns further demonstrate that AB and AP did not form co-crystals but rather produced a mechanically mixed co-particle system.

Table 4 lists the unit cell parameters of the thermally treated products, showing that while they share the same crystal system, their space groups differ. Furthermore, the diffraction peak positions of the three thermally treated products showed no significant overall differences, with only minor variations in intensity, indicating their fundamental structural similarity.

**Figure 3:**
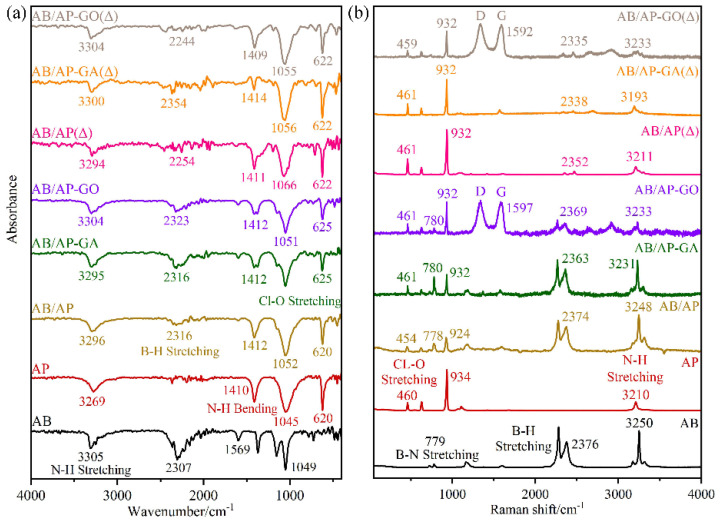
FTIR and Raman spectra of composite particles. (**a**) FTIR (**b**) Raman of Composite Particles.

The FTIR and Raman spectra of the composite were tested, and the results are shown in Figure 3. The characteristic peaks corresponding to each functional group are shown in Table 5 and Table 6.

The characteristic peaks of Cl-O and B-H were found in the three composite samples, which also proved that there were two kinds of substances, AB and AP, present. However, compared with the raw material, the positions of the three characteristic peaks have been shifted to a certain extent, mainly due to the obvious changes in the positions of the stretching vibration peaks of B-H and N-H. It is proven that the hydrogen bond between -NH_3_ and ClO_4_^−^ has been enhanced due to the interaction of N-H···O, which leads to the change of the stretching vibration peak of N-H. The shift of the B-H stretching vibration may be due to the effect of the H-H bond in AB or the formation of a B-H···O hydrogen bond. The characteristic peak shifts of AB/AP-GO and AB/AP-GA were the same, and the D peak and G peak of graphene oxide were found at 1338 cm^−1^ and 1597 cm^−1^. The vibration mode of these three composite particles has been changed because of intermolecular interactions, such as hydrogen bonds, and the intensity and position of the absorption peak have also changed, and it is no longer caused by a single component intermolecular interaction. It was proved that the composite particles were not a simple mechanical mixture, and a new phase was formed, which was different from the mechanical mixture.

The main components of the three heat treatment products are related compounds of amino borane perchlorate. Among them, the stretching vibration peak intensity of ClO_4_^−^ is particularly significant, and there are also relatively weak B-H stretching vibration peaks and bending vibration peaks. It is worth noting that the characteristic peak positions of these three products are shifted compared with AP, which is mainly due to the formation of APB. This process leads to significant changes in the original functional groups and their interactions, leading to the shift of the characteristic vibration peak positions of B-H and N-H. Compared with AB, the B-N stretching vibration peak of the three heat-treated products disappeared completely. Due to the low content of AB, only part of AP participated in the formation of APB during the heat-treatment process. During this process, some cyclo-borane and APB sublimate and precipitate, which reduces the proportion of amino borane in the system. At this stage, the concentration of cyclization products is very low, below the detection limits of both FTIR and Raman spectroscopy, and this leads to the disappearance of the B-N stretching vibration peak. The shift of the characteristic peak position is due to the change in the interaction between the original AB and AP molecules upon generation of APB. The Raman test results are consistent with those of the FTIR results, which proves that the three products contain APB after heat treatment. These derivatives change the interaction between molecules and form a new phase.

To measure the mass calorific value of the materials, the combustion heat of the composite particles was determined through ignition under a 3 MPa oxygen atmosphere. The density of each material was measured using a densitometer, and the corresponding volumetric calorific values were then calculated. The specific experimental data are presented in Table 7.

The energetic properties of the materials were evaluated by measuring the combustion heat of composite particles under a 3 MPa oxygen atmosphere using bomb calorimetry, while the material densities were determined with a densitometer to obtain volumetric heat values (specific data presented in Table 7). The results demonstrate that pure AB achieved the mass-specific heat value of 42.54 kJ·g^−1^ in oxygen, whereas pure AP exhibited a mass-specific heat value of 3.64 kJ·g^−1^.

As shown in Table 7, the theoretical mass combustion heat of the AB/AP mechanical mixture reached 11.42 kJ·g^−1^, while the combustion heats of the three composite particles were relatively close to this value, indicating that the actual molar ratios of AB to AP in the composites were consistent with the theoretical design. However, the unmodified AB/AP exhibited higher combustion heat, demonstrating that the incorporation of graphene-based materials reduced the energetic performance of the composites. The combustion heats of all three thermally treated products showed significant decreases compared to the raw mechanical mixture: AB/AP(Δ) decreased by 15.3%, AB/AP-GA(Δ) by 21.8%, and AB/AP-GO(Δ) by 30.5%. This reduction was primarily attributed to the sublimation of some ammonia borane derivatives [32,33,34,35,36], which led to decreased energetic performance of the system. Nevertheless, the remaining energy performance still maintained a relatively high level, suggesting that the formation of APB [32,33,34,35,36,40] partially compensated for the energy loss. Furthermore, the mass combustion heat data revealed that APB itself possessed relatively high combustion heat. This finding further confirmed that the addition of graphene-based materials negatively impacted the energetic properties of the composite system. The experimental results demonstrated that while graphene modification affected combustion performance, the as-prepared APB derivatives helped maintain considerable energy output in the composites.

The density data in Table 7 reveal that the measured densities of the composite materials were generally higher than their theoretical values, demonstrating that the molecular interactions between AB and AP components extended beyond simple mechanical mixing to include significant intermolecular forces that promoted tight binding. Notably, the unmodified composites exhibited higher densities compared to their modified counterparts, as the incorporation of low-density graphene-based materials increased molecular dispersion within the modified composites, thereby reducing their overall density. After thermal treatment, all materials showed substantial density increases relative to theoretical values: AB/AP(Δ) by 30.8%, AB/AP-GA(Δ) by 26.2%, and AB/AP-GO(Δ) by 24.4%. These enhancements confirmed that the formation of APB during thermal processing altered the intermolecular interactions, leading to significant modifications in product density. Furthermore, the inherently high density of APB itself may have contributed to the observed increases in system density. The thermal treatment significantly enhanced the density of AB/AP composites, effectively addressing the issue of low density inherent to pure AB materials.

### 2.3. Thermal Behavior and Decomposition Kinetics

#### 2.3.1. Non-Isothermal Mass Loss and Heat Flow Properties

**Figure 4:**
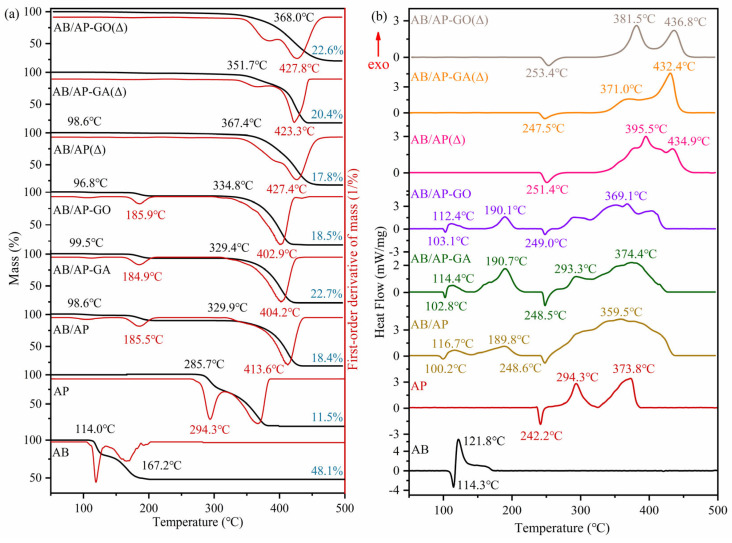
Thermal decomposition of AB, AP, and three composite particles: (**a**) TG-DTG curves and (**b**) DSC curves.

The thermal reactivity of the raw materials AB, AP, and the composite particles was investigated using a differential scanning calorimeter (DSC). The experimental conditions were maintained as follows: argon gas flow rate at 40 mL/min, heating rate of 10 K·min^−1^, and a temperature range from 50 to 500 °C. The resulting TG-DTG and DSC curves are presented in Figure 4, and the thermal decomposition parameters are summarized in Table 8 and Table 9.

As shown in Figure 4, the thermal decomposition of AB occurred in two distinct stages: (i) Between approximately 112 and 130 °C, the first decomposition stage took place (NH_3_-BH_3_ → -[NH_2_-BH_2_]_n_- + H_2_), releasing one hydrogen molecule. AB melted between 97 and 120 °C, indicating that the first decomposition step occurred simultaneously with melting. (ii) In the temperature range of 130–200 °C, the second decomposition stage proceeded (-[NH_2_-BH_2_]_n_- → -[NH=BH]_n_- + H_2_), releasing an additional hydrogen molecule and leaving a residue of 41%. The total heat released during AB decomposition was measured at 616.3 J·g^−1^, consistent with previously reported thermal stability data for AB [32,33,34,35,36,37]. Additionally, an endothermic peak at 242.2 °C was identified, corresponding to the phase transition of AP from orthorhombic to cubic crystal structure. AP decomposition also exhibited two stages: (i) The low-temperature decomposition stage occurred between 274 and 300 °C, with an associated heat release of 361 J·g^−1^. (ii) The high-temperature decomposition stage took place from 300 to 382.2 °C, releasing 631 J·g^−1^ of heat and resulting in a final residue of 11.5%.

The AB/AP composite particles prepared by direct freeze-drying exhibited a two-stage decomposition profile, with each stage corresponding to the decomposition of AB and AP components, respectively. Notably, the low-temperature decomposition of AP disappeared, retaining only its high-temperature decomposition. The initial decomposition temperature of AP in the composite showed a significant increase of 70–80 °C compared to pristine AP. Furthermore, the heat release during AP decomposition in the composite (2997 J·g^−1^) substantially exceeded that of pristine AP (962 J·g^−1^). The residual mass ratio after decomposition of the AB/AP composite particles was measured at 18.4%. Similarly, the modified AB/AP composites only displayed high-temperature decomposition of AP with equivalent temperature elevation (70–80 °C). The decomposition heats for AB/AP-GA (1184 J·g^−1^) and AB/AP-GO (1777 J·g^−1^) composites both surpassed that of pristine AP (962 J·g^−1^). The residual mass ratios after decomposition were determined to be 22.7% and 18.5% for AB/AP-GA and AB/AP-GO composites, respectively.

Comparative analysis revealed that all three types of prepared composite particles induced the appearance of two distinct exothermic peaks during AB decomposition, while eliminating the low-temperature decomposition of AP and retaining only its high-temperature decomposition with significantly elevated decomposition temperatures. This alteration in thermodynamic properties provided clear evidence that the prepared composite particles were not simple mechanical mixtures but rather demonstrated enhanced thermal stability due to the formation of intermolecular interactions. Furthermore, the experimental residue quantities of all three composites (18.4%, 22.7%, and 18.5%) showed close agreement with the theoretically calculated value of 18.8%. This consistency confirmed that the actual AB/AP ratios in the synthesized composites corresponded well with the designed experimental proportions.

Notably, the unmodified AB/AP composite particles exhibited significantly higher heat release compared to their modified counterparts, which aligned consistently with the combustion heat data. Pure AB typically left behind boron–nitrogen polymeric chains and borazine derivatives after decomposition at 200 °C, which retained certain hydrogen storage capabilities but required extremely high temperatures (2000 °C) for complete conversion to boron nitride. According to Refs. [32,33,34,35,36,37,38] and experimental phenomena, we found that the presence of AP fundamentally altered this decomposition pathway. The residual boron–nitrogen compounds participated in redox reactions with oxidative species generated during AP’s high-temperature decomposition, thereby significantly enhancing the exothermic output. In contrast, while the modified composites (AB/AP-GA and AB/AP-GO) still demonstrated increased heat release, their energetic performance was compromised because: (1) the incorporated graphene materials were non-energetic themselves, and (2) they competed for reaction with the oxidative intermediates during AP decomposition. This competition resulted in incomplete oxidation of the boron–nitrogen species, ultimately leading to reduced heat release compared to the unmodified system.

In Figure 4, it can be observed that a portion of the substance had already decomposed before 200 °C, although the overall weight loss ratio remained extremely low (less than 1%). This phenomenon might have been caused by incomplete hyperchlorination of B-N polymers during the dehydrogenation process. Comparative analysis of the three thermal decomposition curves revealed that while the endothermic peak of AP persisted, no low-temperature decomposition of AP was detected in the AB/AP(Δ) sample. However, the characteristic two-stage decomposition of AP was clearly evident in both the AB/AP-GA(Δ) and AB/AP-GO(Δ) samples. Notably, all three heat-treated samples exhibited decomposition temperatures as high as 350 °C, approximately 250 °C higher than the decomposition temperature of the raw AB material, demonstrating the significantly enhanced thermal stability of the composite materials after heat treatment.

The decomposition heat release values of AB/AP(Δ) and AB/AP-GA(Δ) after heat treatment (1098 J·g^−1^ and 1213 J·g^−1^, respectively) were higher than that of pristine AP (962 J·g^−1^). In contrast, AB/AP-GO(Δ) showed a lower decomposition heat release (801 J·g^−1^) compared to AP. Notably, all three samples exhibited residual rates close to 18.8%, suggesting minimal loss of AB through sublimation during the heat treatment process.

Comparative analysis revealed that the thermal stability of all three heat-treated products was significantly enhanced relative to AP. Based on Refs. [32,33,34,35,36,37,38] and experimental phenomena, during heat treatment, only trace amounts of ammonia borane derivatives underwent sublimation. While AB/AP(Δ) showed no low-temperature AP decomposition, both AB/AP-GA(Δ) and AB/AP-GO(Δ) displayed this characteristic. This difference was attributed to the establishment of a new intermolecular interaction system between the APB and AP formed during heat treatment, which fundamentally altered the thermal stability of the products. However, the introduction of graphene-like materials weakened these newly formed intermolecular interactions, thereby allowing the low-temperature decomposition of AP to persist in both AB/AP-GA(Δ) and AB/AP-GO(Δ).

#### 2.3.2. Decomposition Kinetic Parameters and Physical Models

Thermal analysis kinetics is widely employed in the field of energetic materials, enabling the investigation of reaction mechanisms and thermal stability through kinetic parameters [41,42,43,44]. To determine the thermal decomposition activation energy (*E_a_*) of AB/AP composite particles, TG-DSC tests were performed at heating rates of 2.5, 5, 7.5, and 10 K·min^−1^. However, the experimental data obtained at 2.5 K·min^−1^ were unsuitable for calculating kinetic parameters. The DTG curves in Figure 4 reveal two distinct decomposition peaks at approximately 180 °C (Peak I) and 410 °C (Peak II) during AB/AP thermal decomposition. Additionally, calcinated AB/AP exhibited a prominent peak around 420 °C. Molecular dynamics calculations were subsequently conducted for these characteristic peaks using DTG data from different heating rates.

**Figure 5:**
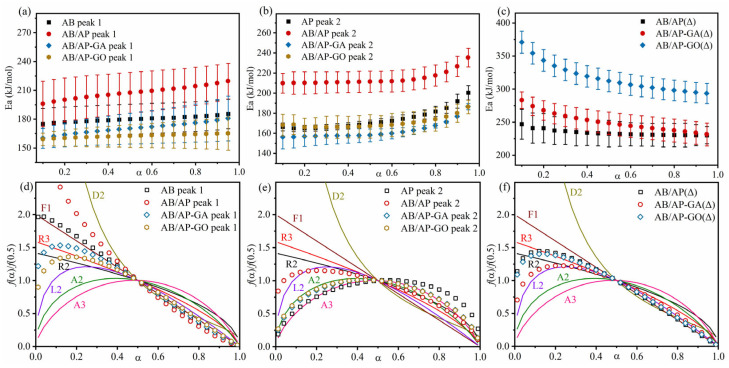
(**a**) The relationship between *E_α_* and *α* during the thermal decomposition of AB and AB/AP composite particles (I) calculated by the Friedman method. (**b**) The relationship between *E_α_* and *α* during the thermal decomposition of AB and AB/AP composite particles (II) calculated by the Friedman method. (**c**) The relationship between *E_α_* and *α* during the thermal decomposition of AB/AP(Δ), AB/AP-GA(Δ), and AB/AP-GO(Δ) calculated by the Friedman method. (**d**) The thermal decomposition models of AP and AB/AP composite particles (I) calculated by the combined kinetic method. (**e**) The thermal decomposition models of AP and AB/AP composite particles (II) calculated by the combined kinetic method. (**f**) The thermal decomposition models of AB/AP(Δ), AB/AP-GA(Δ), and AB/AP-GO(Δ) calculated by the combined kinetic method.

AB/AP systems were further investigated using the joint kinetic method [45] to calculate kinetic parameters and determine the most probable mechanism functions (Figure 5, Table 10). The analysis revealed that at Peak I, AB decomposition followed the F1 model (Unimolecular decay law), while AB/AP exhibited between F1 and D2 model (bidimensional particle shape). The high-temperature decomposition of AP consistently conformed from the A2 to the A3 model. At Peak II, AB/AP followed the L2 model (Random scission), AB/AP-GA and AB/AP-GO exhibited between the A2 and A3 model, confirming the disappearance of AP’s low-temperature decomposition while retaining its high-temperature pathway. Notably, the calcinated products displayed distinct decomposition mechanisms: AB/AP(Δ), AB/AP-GA(Δ), and AB/AP-GO(Δ) all preferentially followed from the L2 to the R3 model (Phase boundary-controlled reaction). These findings indicated that: (1) AB/AP(Δ)’s decomposition mechanism differed significantly from raw AP, and that (2) the simultaneous decomposition of APB and AP under high-temperature conditions substantially modified the reaction mechanism.

The reactive molecular dynamic simulations on thermal decomposition confirmed that AP in the composite particles exclusively underwent high-temperature decomposition, validating the previously proposed decomposition mechanism for AB/AP composites. Based on the theoretical results, the thermal decomposition process revealed distinct stages: (1) AB initially decomposed, releasing hydrogen gas while generating boron–nitrogen polymeric chains and cyclic borazine derivatives. (2) These boron–nitrogen polymers subsequently encapsulated AP particles, altering the intermolecular interactions between AB and AP components. (3) With continued temperature increase, the modified molecular environment caused AP to bypass its characteristic low-temperature decomposition and proceed directly to high-temperature decomposition. Comparative analysis demonstrated significant mechanistic differences between AB/AP(Δ) and pure AP systems. The simultaneous decomposition of thermally generated APB and AP fundamentally modified the reaction pathway, resulting in a distinct decomposition mechanism for AB/AP(Δ).

### 2.4. Isothermal Pyrolysis Products and Decomposition Mechanisms

**Figure 6:**
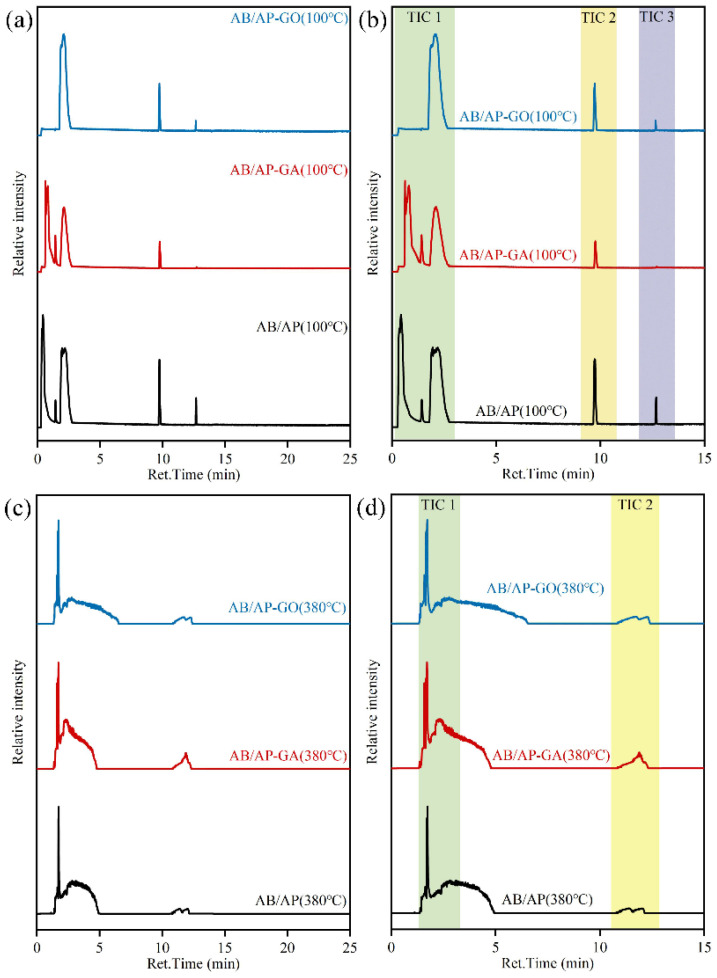
Total Ion Current (TIC) signal at a constant temperature of 100 °C and 380 °C: (**a**) 0–25 min at 100 °C, (**b**) 0–15 min at 100 °C, (**c**) 0–25 min at 380 °C, and (**d**) 0–15 min at 380 °C.

Pyrolysis GC-MS analysis was conducted to characterize the gaseous pyrolysis products of the three composite particle types. To simulate the heat treatment process, the pyrolysis chamber temperature was initially maintained at 100 °C with a heating rate of 20 °C·min^−1^, yielding the TIC curves presented in Figure 6. Subsequently, the chamber temperature was elevated to 380 °C at the same heating rate for additional gas-phase product analysis, with corresponding TIC results shown in Figure 6. Since the predominant peaks in the TIC profiles appeared within the first 15 min, mass spectral analysis focused on the strong peak regions (TIC 1 and TIC 2) during this initial period. The time-resolved pyrolysis mass spectra are displayed in Figure S1, while the identified gaseous products are summarized in Table 11.

Comparative analysis of the TIC profiles revealed distinct differences in the strong peak positions among the three composite particles during 100 °C pyrolysis. Mass spectral characterization demonstrated that while peak positions varied, the gaseous products at these TIC strong peaks were essentially identical across all three materials. Specifically: N_2_ and O_2_ were detected in AB/AP and AB/AP-GA composites between 0.30 and 1.50 min. Cyclotriborazane derivatives appeared at 1.90–2.20 min. Modified composites exhibited N_2_ and APB signatures at 9.70–9.80 min, while AB/AP showed perchloric acid and APB. During 12.60–12.80 min, N_2_ and APB were consistently observed in modified composite products.

Comparing all gas products’ mass spectra, it can be seen that at 100 °C, AP did not reach the decomposition temperature, and pure AP would not produce any gas products under heating conditions at 100 °C. However, H_2_O was found in the gas-phase products N_2_, O_2_, H^35^Cl, N_2_O, and nitric acid [46] substances were also found, and the formation of cyclic compounds such as cyclotriborazane and APB was also discovered during the heat treatment process.

Comparative analysis of the TIC curve strong peak positions revealed that AB/AP-GO exhibited no detectable peaks between 0.30 and 1.50 min, indicating that graphene oxide incorporation significantly altered the decomposition pathway of AB/AP composites during heat treatment. Unlike AB/AP and AB/AP-GA, which sequentially released O_2_ and N_2_, AB/AP-GO directly co-generated multiple products at 1.90 min, including N_2_, nitric acid [46], and cyclotriborazane derivatives [35,36,37,38,39,40]. These findings demonstrated that graphene oxide addition modified the reaction mechanism by suppressing AP’s characteristic decomposition into O_2_ and N_2_ in the composite system.

During the heat treatment of AB/AP composites, AB decomposition produced H_2_, cyclotriborazane derivatives, and boron–nitrogen polymeric species. The presence of AP induced significant chemical interactions, where the highly electronegative ClO_4_^−^ ions promoted the formation of NH_3_BH_2_^+^ species from AB, which subsequently combined with ClO_4_^−^ to form APB [40]. Concurrently, boron–nitrogen polymers generated from AB decomposition also reacted with ClO_4_^−^ to produce additional APB. This process caused partial AP to undergo redox reactions through ClO_4_^−^ loss, while the remaining unreacted AP and newly formed APB collectively constituted the final heat-treated product.

Comparative analysis of the TIC profiles at 380 °C pyrolysis revealed nearly identical peak positions among the three composite particles. Mass spectral characterization confirmed similar gaseous product distributions at corresponding TIC peaks. Specifically: NH_3_, N_2_, N_2_O, and cyclotriborazane derivatives were detected in all three composites between 1.58 and 1.59 min. Additional hypochlorous acid and nitric acid appeared at 1.70–1.75 min. During 2.30–2.85 min, NH_3_, H_2_O, and HCl were consistently identified in the decomposition products of all materials.

Comparative analysis of the mass spectra for all gaseous products revealed that the three composite materials generated essentially identical gas species during thermal cracking. Notably, the two modified AB/AP composites (AB/AP-GA and AB/AP-GO) produced additional CO and CO_2_, demonstrating that oxidative species (particularly O_2_) derived from AP decomposition oxidized the graphene-based additives during the process.

Gas-phase products N_2_, O_2_, H^35^Cl, N_2_O, and nitric acid [46] substances both originated from the gas products of the AP thermal cracking process [46]. Due to the stable presence of ^35^Cl and ^37^Cl in the natural chlorine element, the industrial grade ammonium perchlorate used may contain these two chlorine isotopes. The macromolecular compounds were B_3_N_3_H_6_, which were gaseous products produced during the thermal decomposition of AB [32,33,34,35,36]. These substances still had a certain hydrogen storage capacity, but they would sublime into gas above 80 °C [34,40].

Comparative analysis of the TIC curves and TG-DSC data reveals that during heating, AB and AP in the AB/AP composite underwent separate decomposition processes. The thermal decomposition occurred in two distinct stages: Between 100 and 200 °C, AB decomposed to release hydrogen gas while generating boron–nitrogen polymeric chains and borazine derivatives, both of which maintained hydrogen storage capacity. These boron–nitrogen polymers subsequently encapsulated AP particles, significantly altering the AB–AP intermolecular interactions. Upon further heating to 350 °C, the modified molecular environment caused AP to bypass its characteristic low-temperature decomposition and proceed directly to high-temperature decomposition. This process generated oxidizing gases (H_2_O, N_2_, O_2_, HCl) that reacted exothermically with the boron–nitrogen polymers, thereby enhancing the overall heat release during AP decomposition in the composite system.

## 3. Discussion

**Figure 7:**
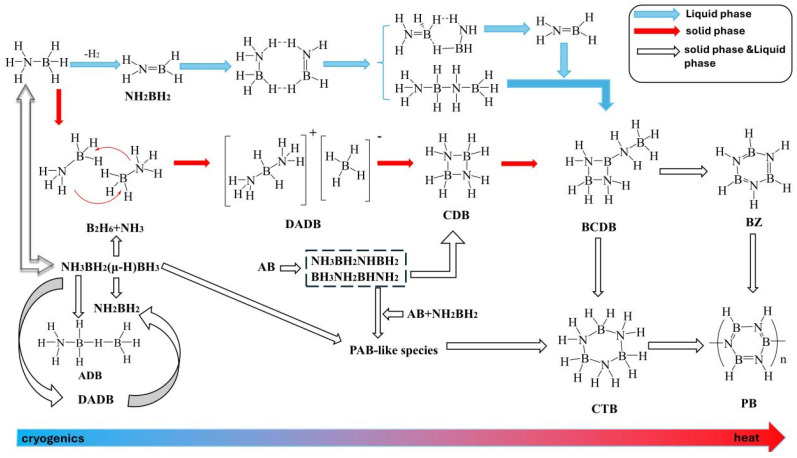
The mechanism of AB thermal decomposition.

The thermal decomposition process of pure AB can be summarized from previous studies (see Figure 7): the initial stage of AB decomposition forms BH_2_, BH, BH_3_, etc. These compounds are subsequently converted into DADB, chain-like products, and H_2_ [32,33,34,35,36]. Chainlike substances undergo self-catalytic reactions to form PABs. Under the catalysis of AB and NH_2_BH_2_, PAB would proliferate to form acyclic PABs or undergo cyclization to form BZ and PBZ. At the same time, the open-loop reaction of DADB gradually converts it into BZ, ultimately producing PBZ.

**Figure 8:**
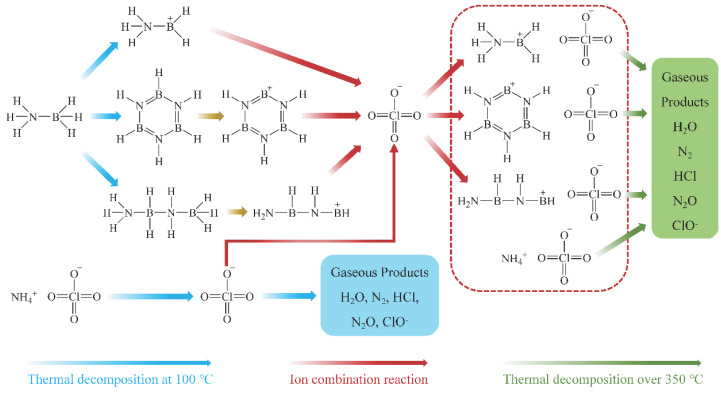
Thermal decomposition process of AB/AP composite particles.

Based on gas-phase product analysis from thermal cracking at 100 °C and 380 °C, the thermal decomposition process of AB/AP composite particles can be summarized (see Figure 8). During calcination at 100 °C, the binding between AP and AB initiates the following reactions: AB releases H_2_ while forming borazine (BZ) and boron–nitrogen polymeric chains. The highly electronegative ClO_4_^−^ from AP promotes NH_3_BH_2_^+^ formation, which combines with ClO_4_^−^ to yield APB [40]. Boron–nitrogen polymers and BZ from AB decomposition further react with ClO_4_^−^ through dehydrogenation to produce additional APB. Concurrently, partial AP undergoes redox reactions via ClO_4_^−^ loss, with the remaining AP and newly formed APB constituting the heat-treated product. Upon further heating above 350 °C, modified intermolecular interactions induce two parallel processes: direct high-temperature decomposition of AP, and oxidative decomposition of APB (major products: 4NH_3_BH_2_ClO_4_ → N_2_↑ + 4HCl↑ + 2B_2_O_3_ + N_2_O↑ + O_2_↑ + 7H_2_O↑ + H_2_↑).

High-density AB/AP composite particles were successfully synthesized through direct freeze-drying at a 1:1 molar ratio (AB: AP). The formation of novel intermolecular interactions increased the composite density by 26.4% compared to mechanical mixtures, while simultaneously eliminating AP’s low-temperature decomposition and raising its high-temperature decomposition onset by 80 °C. This method effectively resolved AB’s inherent low-density limitation.

Subsequent vacuum calcination of the composites produced AB/AP(Δ), which demonstrated superior thermal stability with a decomposition temperature 250 °C higher than pure AB and achieved a density of 1.6604 g/cm^3^. APB in AB/AP(Δ) overcomes the limitations of low density and thermal instability of AB, and has the potential to be used as a high-energy-density additive for high-burning-rate solid propellant.

## 4. Materials and Methods

### 4.1. Materials

Ammonia borane (NH_3_BH_3_, AB) was purchased from Shanghai Aladdin Biochemical Technology Co., Ltd. Ammonium perchlorate (NH_4_ClO_4_, AP) was obtained from Henan Nayu Co., Ltd. Graphene (GA) was acquired from Shenzhen Hongdachang Technology Evolution Co., Ltd. Graphene oxide (GO) was procured from Nanjing Jicang Reagent Co., Ltd. Deionized water was sourced from Tianjin Fuyu Fine Chemical Co., Ltd.

### 4.2. Preparation of AB/AP Composites

#### 4.2.1. Mechanical Mixtures of AB and AP

All AB/AP composites were prepared using the direct freeze-drying method. The preparation process is illustrated in Figure S2.

AB/AP: AB and AP were weighed in a molar ratio of 1:1 and dissolved in 20 mL of deionized water at room temperature. After thorough stirring to ensure complete dissolution, the solution was subjected to vacuum freeze-drying to obtain crystalline products. The yield was 99.7%.

In order to decrease the mechanical sensitivity of AB/AP composites, the carbon-modified AB/AP: 2 wt% of graphene (GA) or graphene oxide (GO) was dispersed in 20 mL of deionized water and ultrasonicated for 1 h. Subsequently, AB and AP were weighed in a molar ratio of 1:1 and dissolved in the ultrasonically dispersed solution at room temperature. After thorough stirring to ensure complete dissolution, the solution was vacuum freeze-dried to remove water and obtain crystalline products. The yield was 99.5% for both. The composite particles with added GA were designated as AB/AP-GA, and those with added GO were designated as AB/AP-GO.

AB/AP mechanical mixture: AB and AP were weighed in a molar ratio of 1:1 and placed in an agate mortar. A small amount of deionized water was added to moisten the mixture, which was then thoroughly ground. After the water evaporated, the mechanical mixture was obtained.

#### 4.2.2. Calcinated Composites of AB and AP

The AB/AP, AB/AP-GA, and AB/AP-GO composites were placed in a vacuum drying oven and calcined at 100 °C for 2 h under vacuum to prepare high-density, thermally stable ammonium perchlorate borane (APB). The heat-treated AB/AP, AB/AP-GA, and AB/AP-GO were designated as AB/AP(Δ), AB/AP-GA(Δ), and AB/AP-GO(Δ), respectively. The preparation process is illustrated in Figure S3.

#### 4.2.3. Characterizations and Theory

The morphology and microstructure of AB/AP composites and heat-treated AB/AP were characterized using scanning electron microscopy (SEM, Hitachi Regulus SU8230) at an acceleration voltage of 15.0 kV. The combustion heat was recorded by the ZDHW-HN9000A calorimeter. The combustion test was performed with 0.2 g of raw material in a 30 mL confined space, under an oxygen pressure of 3 MPa. The chemical bond structures of AB/AP composites and heat-treated AB/AP were investigated using Fourier transform infrared spectroscopy (FTIR, Brucker Corporation TENSOR II) and Raman spectroscopy (Thermal Fisher Scientific Dxr 2Xi). The thermal reactivity of AB/AP composites and calcinated AB/AP was evaluated by thermogravimetry-differential scanning calorimetry (TG-DSC) using a NETZSCH STA 449 simultaneous thermal analyzer under an argon atmosphere with a gas flow rate of 50 mL·min^−1^. The composites were heated within a temperature range of 50–500 °C at heating rates of 2.5, 5, 7.5, and 10 K·min^−1^ to calculate their decomposition kinetics. The gaseous products from the thermal reaction of AB/AP composites and calcinated AB/AP were analyzed using pyrolysis-gas chromatography/mass spectrometry (Pyro-GC/MS, Frontier EGA/PY-3030D). The pyrolysis chamber temperature was set to 380 °C to study the pyrolysis products and to 100 °C to simulate the heat treatment environment, with a heating rate of 20 K·min^−1^. The structures of AB/AP composites and heat-treated AB/AP were characterized using X-ray diffraction (XRD, Rigaku Ultima IV).

## 5. Conclusions

The AB/AP composite particles were prepared using a direct freeze-drying method, followed by thermal treatment to obtain calcinated AB/AP with enhanced thermal stability and density. The reaction mechanism of the thermal treatment process was investigated through pyrolysis gas-phase product analysis. The main findings were as follows:

High-density AB/AP composite particles with a 1:1 molar ratio were successfully prepared via direct freeze-drying. The establishment of novel intermolecular interactions between AB and AP increased the composite density by 26.4% compared to mechanical mixtures. The composite particles retained only AP’s high-temperature decomposition, with an 80 °C higher initial decomposition temperature than pure AP, effectively addressing AB’s low-density limitation.

Three types of composite particles were heated in a vacuum oven at 100 °C for 2 h, yielding oxygen/fuel-integrated APB with superior thermal stability. The thermal decomposition mechanism study revealed that: AB/AP initially decomposed during heat treatment, producing borazine derivatives and boron–nitrogen polymeric chains; AP presence facilitated NH_3_BH_2_^+^ formation, which combined with ClO_4_^−^ to generate APB; and partial AP underwent redox reactions through ClO_4_^−^ loss, with the remaining AP and newly formed APB constituting AB/AP(Δ). Upon further heating to 350 °C, modified intermolecular interactions induced simultaneous AP high-temperature decomposition and APB redox reactions, generating substantial gaseous products. However, carbon additives remained non-reactive during heat treatment, forming heterogeneous interfaces with APB and AP that weakened molecular interactions and reduced thermal stability in modified composites. APB demonstrated remarkable thermal stability (250 °C higher decomposition temperature than AB) and achieved a density of 1.6604 g/cm^3^, overcoming AB’s limitations of low density and poor thermal stability. These properties establish APB as a promising high-energy-density additive for high-burning-rate solid propellants.

## Figures and Tables

**Table 1:** Composition of composite particles.

Weight (%)	AB	AP	GA	GO
AB/AP	50	50	-	-
AB/AP-GA	49	49	2	-
AB/AP-GO	49	49	-	2

**Table 2:** Preparation process of calcinated AB/AP.

Calcinated AB/AP	Preparation Process
AB/AP(Δ)	Calcinate AB/AP at 100 °C for two h
AB/AP-GA(Δ)	Calcinate AB/AP-GA at 100 °C for two h
AB/AP-GO(Δ)	Calcinate AB/AP-GO at 100 °C for two h

**Table 3:** Elemental analysis test results.

Composites		N (%)	C (%)	H (%)	O (%)
Mechanical mixture AB/AP	Experimental	18.10	0	7.30	42.65
	Theoretical	18.87	0	6.74	43.13
AB/AP	Experimental	18.02	0	6.60	42.66
	Theoretical	18.87	0	6.74	43.13
AB/AP-GA	Experimental	17.83	1.54	6.46	42.34
	Theoretical	18.50	1.98	6.61	42.28
AB/AP-GO	Experimental	18.60	0.88	6.84	41.46
	Theoretical	18.62	0.80	6.65	43.09
AB/AP(Δ)	Experimental	10.54	0	3.30	43.21
	Theoretical	18.87	0	6.74	43.13
AB/AP-GA(Δ)	Experimental	13.12	1.63	4.23	42.35
	Theoretical	18.50	1.98	6.61	42.28
AB/AP-GO(Δ)	Experimental	13.36	0.82	3.95	41.63
	Theoretical	18.62	0.80	6.65	43.09

**Table 4:** Unit cell parameters of composite particles.

	AB	AP	Mechanical Mixture AB/AP	AB/AP	AB/AP-GA	AB/AP-GO	AB/AP(Δ)	AB/AP-GA(Δ)	AB/AP-GO(Δ)
Crystall System	Orthorhombic	Orthorhombic	Orthorhombic	Monoclinic	Monoclinic	Monoclinic	Orthorhombic	Monoclinic	Monoclinic
Space Group	Cmc2_1_	Pnma	Pccn	P2	P2	C2	Pccn	P2	P2_1_/C
*a*/Å	12.668	9.226	11.716	6.906	11.516	8.956	15.896	6.773	14.003
*b*/Å	10.825	5.815	13.329	10.796	11.678	8.147	14.437	11.474	13.195
*c*/Å	8.183	7.456	9.850	6.062	8.417	10.021	11.936	19.230	6.637
*α*/deg	90.0	90.0	90.0	90.0	90.0	90.0	90.0	90.0	90.0
*β*/deg	90.0	90.0	90.0	123.5	137.6	113.5	90.0	99.2	104.0
*γ*/deg	90.0	90.0	90.0	90.0	90.0	90.0	90.0	90.0	90.0
*Vol.*/Å^3^	1122.2	400.0	1538.1	376.8	763.4	670.6	2739.2	1475.1	1190.0

**Table 5:** FTIR of AB, AP, and AB/AP composites.

cm^−1^	AB	AP	AB/AP	AB/AP-GA	AB/AP-GO	AB/AP(Δ)	AB/AP-GA(Δ)	AB/AP-GO(Δ)
ClO_4_^−^ Stretching	—	620	620	625	620	622	622	622
	—	1045	1052	1052	1051	1066	1056	1055
N-H Stretching	3305	3269	3296	3295	3304	3294	3300	3304
N-H Bending	1371	—	—	1371	1375	—	—	—
	—	1410	1412	1412	1412	1411	1414	1409
	1569	—	1597	1597	1597	—	—	—
B-H Stretching	2307	—	2316	2316	2323	2254	2354	2244
B-H Bending	1149	—	—	—	—	—	—	—
	1049	—	1148	1149	1146	1195	1191	1189

**Table 6:** Raman spectroscopy of AB, AP, and AB/AP composites.

cm^−1^	AB	AP	AB/AP	AB/AP-GA	AB/AP-GO	AB/AP(Δ)	AB/AP-GA(Δ)	AB/AP-GO(Δ)
Cl-O Stretching	—	460	454	461	461	622	622	622
	—	629	625	625	630	—	—	—
	—	934	924	932	932	1066	1056	1055
N-H Stretching	3250	3210	3248	3231	3233	3294	3300	3304
B-H Stretching	2376	—	2374	2363	2369	2352	2338	2335
B-N Stretching	779	—	778	780	780	—	—	—
D(Disordered State)	—	—	—	—	1338	—	—	1338
G(Graphitic State)	—	—	—	—	1597	—	—	1592

**Table 7:** Density and heat of combustion of composite particles.

Atmosphere	Materials	Mass Heat of Reaction (kJ·g^−1^)	Density (g·cm^−3^)	Volumetric Heat of Reaction (kJ·cm^−3^)
O_2_	AB	42.54	0.7581	32.25
O_2_	AP	3.64	1.9502	7.10
O_2_	Mechanical mixture AB/AP	11.42	1.2693	14.50
O_2_	AB/AP	10.44	1.6047	16.75
O_2_	AB/AP-GA	10.18	1.5375	15.65
O_2_	AB/AP-GO	10.03	1.5176	15.22
O_2_	AB/AP(Δ)	9.67	1.6604	16.07
O_2_	AB/AP-GA(Δ)	8.93	1.6021	14.31
O_2_	AB/AP-GO(Δ)	7.94	1.5796	12.54

**Table 8:** The characteristic TG-DTG parameters of the thermal decomposition for AB, AP, and three composite particles.

Composites	TG Curves		DTG Curves			
	*T*_i_ (°C)	*ML* (%)	*T*_p1_ (°C)	*T*_p2_ (°C)	*T*_p3_ (°C)	*T*_p4_ (°C)
AB	114.0 149.3	21.8 30.1	119.7	167.2	—	—
AP	285.7 333.5	29.1 59.4	—	—	294.3	367.2
AB/AP	98.6 174.1 365.4	2.1 16.3 63.2	—	185.5	—	413.6
AB/AP-GA	99.5 174.8 363.2	0.7 6.5 70.1	—	184.9	—	404.2
AB/AP-GO	96.8 175.1 365.5	0.9 5.476.1	—	185.9	—	402.9
AB/AP(Δ)	367.4	82.2	—	—	—	427.4
AB/AP-GA(Δ)	351.7	79.6	—	—	—	423.3
AB/AP-GO(Δ)	368.0	77.4	—	—	—	427.8

**Table 9:** The characteristic DSC parameters of the thermal decomposition for AB, AP, and three composite particles.

Composites	DSC Curves							
	Endothermic Peaks				Exothermic Peaks			
	*T*_p2_ (°C)	Δ*H*_1_ (J·g^−1^)	*T*_p2_ (°C)	Δ*H*_2_ (J·g^−1^)	*T*_p3_ (°C)	Δ*H*_3_ (J·g^−1^)	*T*_p4_ (°C)	Δ*H*_4_ (J·g^−1^)
AB	114.3	88.5	—	—	121.5	616.3	—	—
AP	—	—	242.2	64.6	—	—	294.3 373.8	361.2 630.8
AB/AP	100.2	11.1	248.6	37.2	116.7 189.8	63.7 317.6	359.5	2996.6
AB/AP-GA	102.8	5.8	248.5	40.6	114.4 190.7	40.4 304.1	374.4	1184.2
AB/AP-GO	103.1	6.8	249.0	31.1	112.4 190.1	53.6 165.5	369.1	1777.2
AB/AP(Δ)	—	—	251.4	74.0	—	—	434.9	1098.3
AB/AP-GA(Δ)	—	—	247.5	67.6	—	—	432.4	1212.8
AB/AP-GO(Δ)	—	—	253.4	66.5	—	—	436.8	800.8

**Table 10:** The decomposition kinetic parameters of the composite particles based on non-isothermal DTG curves.

Samples	Combined Kinetic Method				Friedman Method		Kissinger Method		
	*m*	*n*	*E_a(1)_*/ kJ·mol^−1^	*cA*/ min^−1^	*E_a(2)_*/ kJ·mol^−1^	*r*	*E_a(3)_*/ kJ·mol^−1^	Log *A*	*r*
AB-I	0.029	1.150	187.0 ± 1.4	4.66 × 10^21^	179.8	0.9925	61.7	10.51	0.9968
AP-II	0.521	0.424	171.5 ± 1.0	3.97 × 10^12^	174.0	0.9994	41.9	6.71	0.9961
AB/AP-I	−0.123	1.251	218.8 ± 1.6	8.32 × 10^23^	207.0	0.9894	58.3	9.86	0.9970
AB/AP-II	0.158	0.608	228.6 ± 3.1	1.14 × 10^17^	214.2	0.9896	42.1	6.76	0.9980
AB/AP-GA-I	0.158	1.152	179.3 ± 1.4	2.24 × 10^19^	169.8	0.9910	58.2	9.84	0.9972
AB/AP-GA-II	0.487	0.608	165.2 ± 0.9	1.90 × 10^12^	162.1	0.9982	42.3	6.79	0.9973
AB/AP-GO-I	0.228	1.088	167.7 ± 1.3	9.33 × 10^17^	162.1	0.9946	58.3	9.86	0.9967
AB/AP-GO-II	0.466	0.607	175.5 ± 2.3	1.77 × 10^13^	170.2	0.9915	42.4	6.82	0.9974
AB/AP(Δ)	0.157	1.052	244.2 ± 2.5	7.01 × 10^17^	235.7	0.9852	41.6	6.66	0.9982
AB/AP-GA(Δ)	0.269	0.953	253.6 ± 1.7	3.74 × 10^18^	252.9	0.9958	41.5	6.65	0.9982
AB/AP-GO(Δ)	0.160	1.000	317.1 ± 1.6	1.47 × 10^23^	321.8	0.9975	41.4	6.62	0.9983

**Table 11:** The molecular formulas corresponding to the gaseous products from the pyrolysis mass spectrometry of the three composite particles at 100 °C and 380 °C.

*m*/*z*	Samples					
	AB/AP 100 °C	AB/AP-GA 100 °C	AB/AP-GO 100 °C	AB/AP 380 °C	AB/AP-GA 380 °C	AB/AP-GO 380 °C
14	N	N	—	—	—	—
17	—	—	—	NH_3_	NH_3_	NH_3_
18	H_2_O	H_2_O	—	H_2_O	H_2_O	H_2_O
28	N_2_	N_2_, CO	N_2_, CO	N_2_	N_2_, CO	N_2_, CO
30	—	—	—	NO	NO	NO
32	O_2_	O_2_	—	O_2_	O_2_	O_2_
35	—	—	—	—	^35^Cl	—
36	—	—	—	H^35^Cl	H^35^Cl	H^35^Cl
38	—	—	—	—	H^37^Cl	—
44	N_2_O	N_2_O, CO_2_	N_2_O, CO_2_	N_2_O	N_2_O, CO_2_	N_2_O, CO_2_
51–53	(BH_x_NH_y_)_2_	(BH_x_NH_y_)_2_	(BH_x_NH_y_)_2_	(BH_x_NH_y_)_2_	(BH_x_NH_y_)_2_	(BH_x_NH_y_)_2_
62	NO_3_^−^	NO_3_^−^	NO_3_^−^	NO_3_^−^	NO_3_^−^	NO_3_^−^
63	HNO_3_	HNO_3_	HNO_3_	HNO_3_	HNO_3_	HNO_3_
76–81	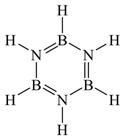					
100	H^35^ClO_4_	—	H^35^ClO_4_	—	—	—
101	^37^ClO_4_^−^	—	—	—	—	—
102	H^37^ClO_4_	—	—	—	—	—
129–133	NH_3_BH_2_^37^ClO_4_	NH_3_BH_2_^37^ClO_4_	NH_3_BH_2_^37^ClO_4_	—	—	—
155–159	NH_2_BHNHBH ^37^ClO_4_^−^	NH_2_BHNHBH ^37^ClO_4_^−^	NH_2_BHNHBH ^37^ClO_4_^−^	—	—	—

## Data Availability

The data presented in this study are available upon request from the corresponding author.

## Supplementary Materials

The following supporting information can be downloaded at: https://www.mdpi.com/article/10.3390/molecules30132680/s1, Figure S1: The mass spectra of pyrolysis of AB/AP composite particles and calcinated AB/AP at 100 °C and 380 °C; Figure S2: Synthesis of AB/AP composites; Figure S3: Synthesis of calcinated AB/AP crystals.
