# Supplementary material for: Thermal Interaction Mechanisms of Ammonium Perchlorate and Ammonia Borane
Source: Molecules. 2025 Jun 20;30(13):2680. doi: 10.3390/molecules30132680 (PMC12251061; doi:10.3390/molecules30132680)
Supplement: Supplementary file 1 [file molecules-30-02680-s001.zip › molecules-3612808-supplementary.pdf]

Supporting Information

for

Thermal Interaction Mechanisms of Ammonium Perchlorate and Ammonia Borane

Yunlong Zhang [1], Rui Pu [1], Shaoli Chen [1] and Qilong Yan [1,*]

1. *National Key Laboratory on Solid Rocket Propulsion, Northwestern Polytechnical University, Xi'an 710072, China*

*Correspondence: qilongyan@nwpu.edu.cn; Tel. 029-88494031.

S1. General methods

All purchased reagents are analytically pure and used without further purification. Decomposition temperatures were measured on NETZSCH STA 449 simultaneous thermal analyzer (NETZSCH Scientific Instruments Trading (Shanghai) Ltd, Germany) with a scan rate of 10 K·min^{-1}. The PXRD patterns were recorded on a Rigaku SmartLab SE diffractometer (Cu−Kα radiation). SEM images were obtained on a ZEISS EVO10 MA field-emission scanning electron microscope at an electric voltage of 10 KV. The combustion calorific value was recorded by the ZDHW−HN9000A calorimeter, respectively.

S2. Mass Spectra of Pyrolysis

Pyro GC-MS was used to analyze the pyrolysis gas phase products of three types of composite particles, with a pyrolysis tank temperature set at 100 ℃ to reproduce the heat treatment process. The heating rate is 20 ℃·min^{-1}. At the same time, the temperature of the cracking tank was set to 380 ℃, and the heating rate was 20 ℃·min^{-1} for gas-phase product analysis. The pyrolysis mass spectra of each time period are shown in Figure S1.

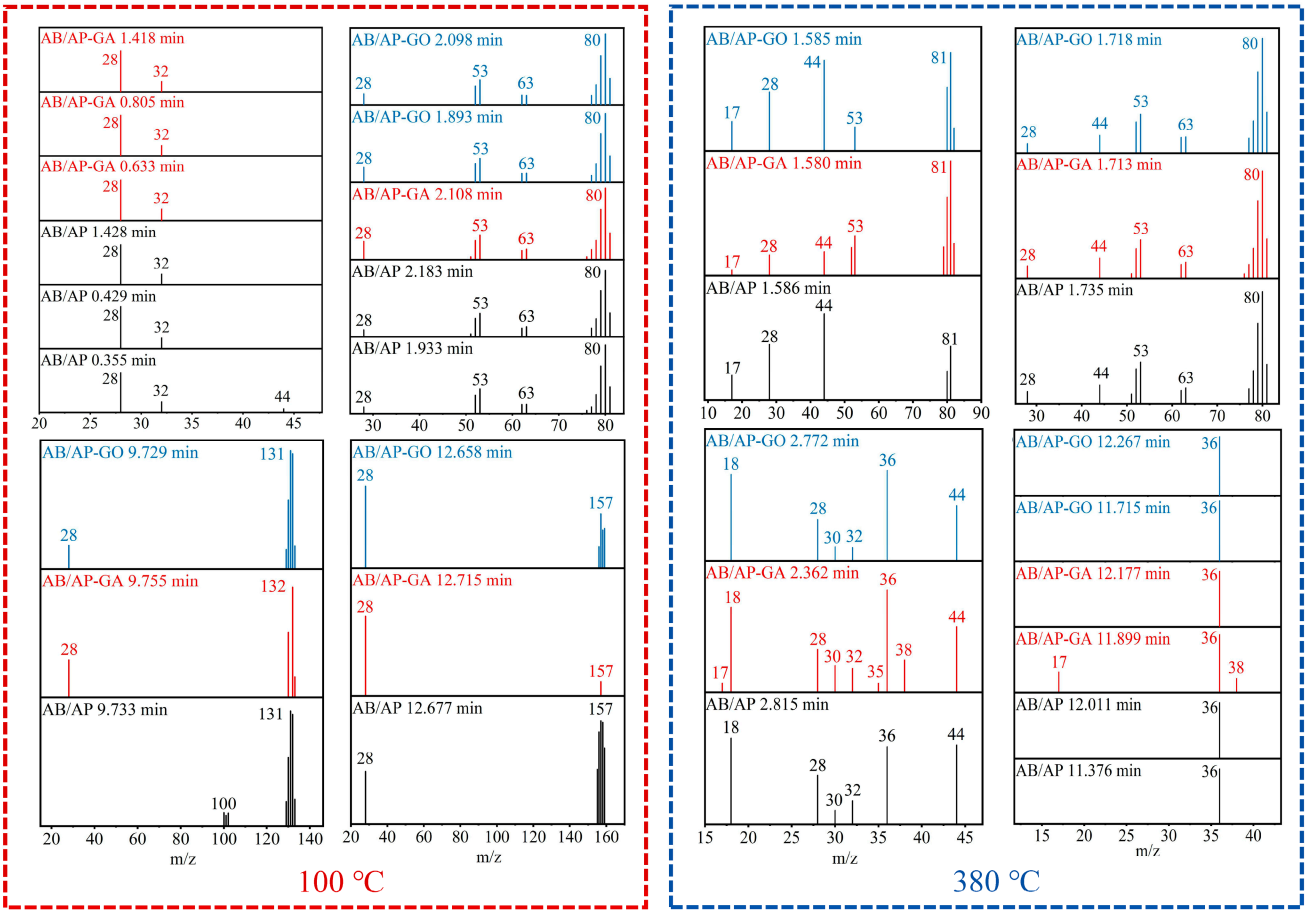

Figure S1. The mass spectra of pyrolysis of AB/AP composite particles and calcinated AB/AP at 100 ºC and 380 ºC.

S3. Synthesis

S3.1 Synthesis of AB/AP composites

All AB/AP composites were prepared using the direct freeze-drying method. The preparation process is illustrated in Figure S2.

AB/AP: AB and AP were weighed in a molar ratio of 1:1 and dissolved in 20 mL of deionized water at room temperature. After thorough stirring to ensure complete dissolution, the solution was subjected to vacuum freeze-drying to obtain crystalline products. The yield was 99.7 %.

In order to decrease the mechanical sensitivity of AB/AP composites, the carbon-modified AB/AP: 2 wt% of Graphene (GA) or graphene oxide (GO) was dispersed in 20 mL of deionized water and ultrasonicated for 1 hour. Subsequently, AB and AP were weighed in a molar ratio of 1:1 and dissolved in the ultrasonically dispersed solution at room temperature. After thorough stirring to ensure complete dissolution, the solution was vacuum freeze-dried to remove water and obtain crystalline products. The yield was 99.5 % for both. The composite particles with added GA were designated as AB/AP-GA, and those with added GO were designated as AB/AP-GO.

AB/AP mechanical mixture: AB and AP were weighed in a molar ratio of 1:1 and placed in an agate mortar. A small amount of deionized water was added to moisten the mixture, which was then thoroughly ground. After the water evaporated, the mechanical mixture was obtained.

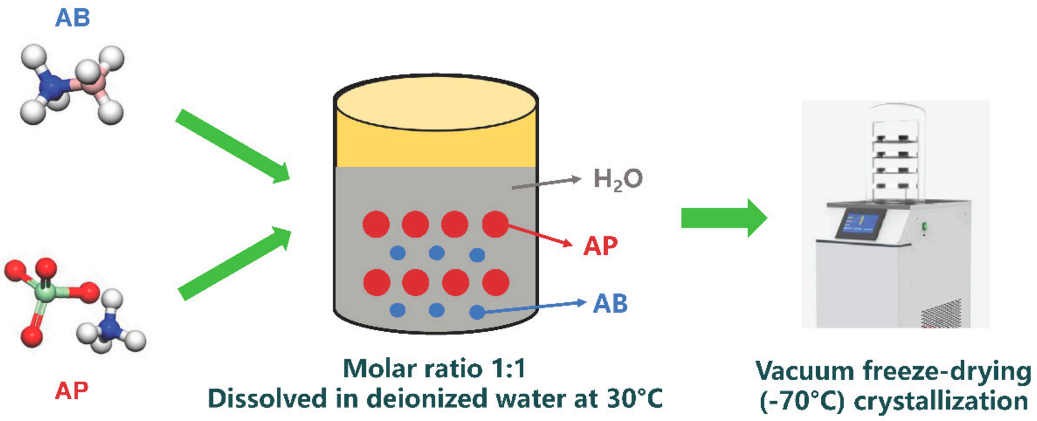

Figure S2. Synthesis of AB/AP composites

S3.2 Synthesis of calcinated AB/AP crystals

The AB/AP, AB/AP-GA, and AB/AP-GO composites were placed in a vacuum drying oven and calcined at 100 °C for 2 hours under vacuum to prepare high-density, thermally stable ammonium perchlorate borane (APB). The heat-treated AB/AP, AB/AP-G, and AB/AP-GO were designated as AB/AP(Δ), AB/AP-GA(Δ), and AB/AP-GO(Δ), respectively. The preparation process is illustrated in Figure S3.

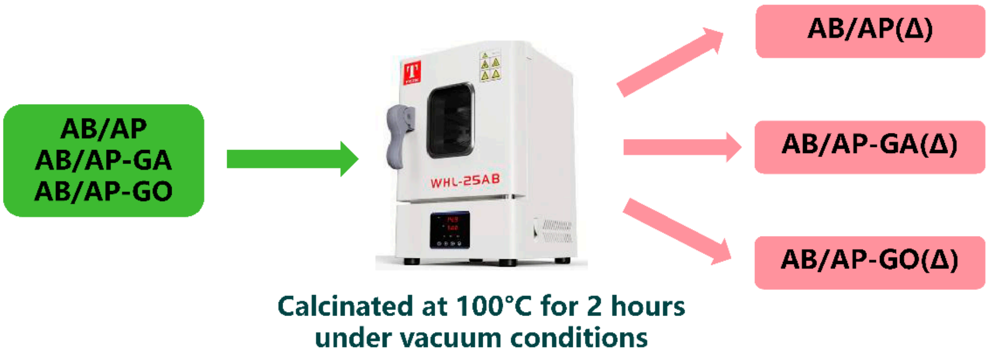

Figure S3. Synthesis of calcinated AB/AP crystals
